# Absolute, weight-based, fixed-dose, or phenotype-guided? Rethinking the meaning of vasopressor dose in critical care

**DOI:** 10.1016/j.aicoj.2026.100094

**Published:** 2026-05-28

**Authors:** Carlos Sanchez-Escalante, Patrick M. Wieruszewski

**Affiliations:** a Critical Care Medicine, Organ Transplant Center of Excellence, King Faisal Specialist Hospital & Research Centre, Riyadh, Saudi Arabia; b Department of Pharmacy, Mayo Clinic, Rochester, Minnesota, United States; c Department of Anesthesiology, Mayo Clinic, Rochester, Minnesota, United States

**Keywords:** Vasopressors, Norepinephrine, Vasopressin, Weight-Based dosing, Phenotype-Guided therapy, Shock

Vasopressor dose is often treated as a technical variable, yet in critical care it functions as a therapeutic intervention, a marker of circulatory failure severity, a trigger for adjunctive therapy, a criterion for refractory shock, and an entry threshold for clinical trials [[1], [2], [3]]. The way dose is expressed is therefore clinically consequential because absolute, weight-based, fixed, and phenotype-guided approaches embed different assumptions about pharmacology, body composition, vascular responsiveness, and disease severity [[5], [6], [7]].

The issue is most apparent with norepinephrine. This distinction is partly pharmacological and partly pragmatic. Catecholamines, including norepinephrine and epinephrine, are short-acting, continuously titrated with broad dose-response ranges. Weight-indexing was adopted to standardize infusion rates and reduce extreme dosing variability across body sizes, although body weight does not necessarily predict vascular responsiveness. Vasopressin differs because adjunctive septic-shock dosing is intended to restore or supplement the relatively deficient vasopressin-pituitary pathway within a narrow dose range, where higher doses may increase ischemic adverse effects rather than provide proportional hemodynamic benefit. Current international guidelines recommend norepinephrine as the first-line vasopressor in septic shock and suggest adding vasopressin rather than further escalating norepinephrine when mean arterial pressure remains inadequate [1]. However, the norepinephrine dose at which clinicians perceive shock as severe, consider adjunctive vasopressors, define refractory shock, or enroll patients into trials depends heavily on how the dose is reported [[2], [3], [4], [5], [6]]. Recent SCCM/ESICM work has shown that even norepinephrine formulation reporting — base molecule versus salt — can alter dose interpretation, clinical comparability, and research thresholds [4]. Importantly, this variation in norepinephrine dose reporting has been shown to impact mortality prediction [5]. More recently, variability between weight-based and non-weight-based norepinephrine dosing units has been highlighted to influence drug exposure, escalation thresholds, prognostication, interpretation of refractory shock definitions, trial comparability, and patient safety [6]. These observations expose a broader principle: vasopressor dose is often used as a physiological signal, but its meaning changes according to the unit chosen.

Weight-based dosing appears individualized because it mathematically adjusts drug infusion to body size, but computational normalization is not necessarily physiological personalization. Indexing a vasopressor to total body weight is meaningful only if body weight reflects the relevant pharmacokinetic compartment, clearance pathway, receptor target, or pharmacodynamic response. This assumption is vulnerable in obesity, where excess adipose tissue may not correspond to the effective distribution volume, vascular target compartment, receptor exposure, or dose-response relationship of short-acting vasoactive drugs [7,8].

In a recent observational study of more than 10,000 patients with septic shock, weight-based dosing altered the association between norepinephrine dose and ICU mortality in obese patients, especially at higher dosages [7]. The mean predicted mortality difference between obese and non-obese patients was 14.5% when norepinephrine was expressed as μg/kg/min, but only 0.3% when expressed as μg/min [7]. These hypothesis-generating data suggest that absolute norepinephrine dose may be a more body mass index–consistent marker of vasopressor burden for severity stratification, prognostication, escalation, or trial comparability, rather than bedside titration. However, this interpretation remains limited by the retrospective design and requires rigorous prospective validation before informing prescriptive dosing recommendations.

In obesity, dividing norepinephrine dosing rate by total body weight may obscure the apparent vasopressor burden without indicating lower shock severity or less pharmacological exposure [7,8]. In critically ill obese patients, automatic total body weight escalation is not appropriate for several supportive-care drugs, including hemodynamic support agents [8]. Therefore, excess adipose tissue may not represent a clinically relevant compartment requiring proportionally greater norepinephrine doses. In this setting, μg/kg/min may create the appearance of personalization while introducing body mass index–dependent misclassification of vasopressor burden and mortality risk.

This does not mean that norepinephrine should be prescribed or titrated blindly according to absolute dose. The central concern is not bedside titration itself, which should remain guided by arterial pressure, hemodynamic response, cardiac output when available, and tissue-perfusion endpoints [1,7]. Rather, the problem arises when norepinephrine dose is repurposed as a severity marker, escalation threshold, trial inclusion criterion, or prognostic variable; in those contexts, weight-based indexing may misclassify obese patients [[5], [6], [7]]. A dose unit that is convenient for infusion adjustment may therefore be inappropriate when repurposed as a marker of disease severity. Phenotype-guided interpretation contextualizes absolute, weight-based, and fixed-dose reporting according to response phenotype, including vasoplegia severity, pressure-flow response, cardiac output or echocardiographic reserve, lactate kinetics, skin perfusion, urine output, acid-base status, body size, and prior catecholamine exposure. These markers help distinguish likely responders from non-responders and clarify whether dose is being used for titration, escalation, severity stratification, prognostication, or trial eligibility (Table 1).

**Table 1:** Absolute, weight-based, fixed-dose, and phenotype-guided approaches to vasopressor dose interpretation.

Approach	What it captures	What it may miss	Clinical implication
Absolute dosing	Total vasopressor delivery per unit time (e.g., mcg/min)	Body-size and pharmacokinetic variability	May be useful for comparing vasopressor burden across BMI groups
Weight-based dosing	Dose normalized to total body weight (e.g., μg/kg/min)	Obesity-related distortion when total body weight does not reflect the vascular target compartment	Useful for infusion adjustment, but potentially misleading as a severity marker
Fixed dosing	Standardized drug delivery (e.g., 0.03 unit/min vasopressin for all)	Heterogeneous physiological response across phenotypes	Fixed delivery should not be interpreted as fixed effect
Phenotype-guided interpretation	Dose interpreted according to response phenotype, drug mechanism, cardiovascular reserve, body size, and hemodynamic response	Requires more clinical information than dose alone	Helps distinguish likely responders from non-responders and aligns dose interpretation with titration, escalation, prognostication, and trial design

Vasopressin illustrates a complementary issue. Unlike norepinephrine, vasopressin is commonly administered as a fixed infusion rate, often 0.03 U/min in septic shock [1,[9], [10], [11], [12]]. This avoids mathematical dilution by total-body-weight indexing, but fixed dosing should not be mistaken for physiological personalization. A fixed infusion standardizes delivery, not response. The hemodynamic effect of vasopressin depends on vascular phenotype, including vasoplegia severity, endogenous vasopressin deficiency, V1a receptor responsiveness, acid–base status, lactate concentration, corticosteroid exposure, endothelial dysfunction, and catecholamine exposure before vasopressin initiation [[9], [10], [11], [12]]. Prior studies have not consistently identified body mass as a determinant of fixed-dose vasopressin response [10,11]. However, Melchers et al. reported that 79% of 200 patients with septic shock met criteria for vasopressin hemodynamic responsiveness; obesity and hyperlactatemia were negatively associated with response, whereas a norepinephrine dose ≥0.30 μg/kg/min was positively associated with response [13]. These findings reinforce that fixed-dose vasopressin does not imply fixed physiological effect. The parallel with norepinephrine is therefore conceptual rather than pharmacologic, as both standardized dose expressions may conceal response heterogeneity, but through different mechanisms.

Other vasopressors reinforce the same principle. Phenylephrine may increase arterial pressure while reducing stroke volume in patients with limited ventricular reserve, whereas angiotensin II responsiveness may depend more on biological responsiveness and early blood pressure response than on body weight [14,15]. These examples emphasize that vasopressor dose cannot be interpreted independently of mechanism of action, cardiovascular reserve, and the biological pathway being targeted.

The central question is therefore not whether vasopressor dose should be reported or interpreted in absolute or weight-based units, but rather which dosing metric is fit for which purpose. For bedside titration, the key issue is whether arterial pressure, flow, and tissue perfusion are satisfactory [1,7]. For severity stratification, the key issue is whether the dosing metric reflects circulatory failure consistently across body size and phenotype [[5], [6], [7]]. For therapeutic escalation, the key issue is whether a threshold identifies the precise moment to add interventions [1,3,9]. For trial enrollment, the key issue is whether the dosing criterion selects comparable patients at baseline [[3], [4], [5], [6],^15^].

In summary, vasopressor dosing is often presented as a technical issue, but it is fundamentally physiological. The same vasopressor dose may represent different levels of pharmacological exposure, circulatory failure, and physiological response depending on the drug, patient phenotype, and clinical purpose [6,7,9,14,15]. Standardization of reporting is necessary, but it is not sufficient. Dose units are not neutral. They require phenotype-guided interpretation.

## Authors’ contributions

Carlos Sanchez-Escalante: Conceptualization, investigation, writing – original draft, writing – review and editing. Patrick M. Wieruszewski: investigation, writing – review and editing.

## Consent for publication

Not applicable.

## Ethics approval and consent to participate

Not applicable.

## Financial support

None.

## Availability of data and materials

Not applicable.

## Declaration of competing interest

Carlos Sanchez-Escalante declares no conflicts of interest. Patrick M. Wieruszewski receives consulting fees from Wolters Kluwer UpToDate.
